# Socioeconomic gaps over time in colorectal cancer survival in England: flexible parametric survival analysis

**DOI:** 10.1136/jech-2021-216754

**Published:** 2021-05-28

**Authors:** Mari Kajiwara Saito, Manuela Quaresma, Helen Fowler, Sara Benitez Majano, Bernard Rachet

**Affiliations:** 1 Inequalities in Cancer Outcomes Network, Department of Non-Communicable Disease Epidemiology, London School of Hygiene & Tropical Medicine, London, UK; 2 Department of Gastroenterology, IMS Tokyo Katsushika General Hospital, Tokyo, Japan; 3 Department of Non-Communicable Disease Epidemiology, London School of Hygiene & Tropical Medicine, London, UK

**Keywords:** healthcare disparities, neoplasms by site, health inequalities

## Abstract

**Background:**

Despite persistent reports of socioeconomic inequalities in colorectal cancer survival in England, the magnitude of survival differences has not been fully evaluated.

**Methods:**

Patients diagnosed with colon cancer (n=68 169) and rectal cancer (n=38 267) in England (diagnosed between January 2010 and March 2013) were analysed as a retrospective cohort study using the National Cancer Registry data linked with other population-based healthcare records. The flexible parametric model incorporating time-varying covariates was used to assess the difference in excess hazard of death and in net survival between the most affluent and the most deprived groups over time.

**Results:**

Survival analyses showed a clear pattern by deprivation. Hazard ratio of death was consistently higher in the most deprived group than the least deprived for both colon and rectal cancer, ranging from 1.08 to 1.17 depending on the model. On the net survival scale, the socioeconomic gap between the most and the least deprived groups reached approximately −4% at the maximum (−3.7%, 95% CI −1.6 to −5.7% in men, −3.6%, 95% CI −1.6 to −5.7% in women) in stages III for colon and approximately −2% (−2.3%, 95% CI −0.2 to −4.5% in men, −2.3%, 95% CI −0.2 to −4.3% in women) in stage II for rectal cancer at 3 years from diagnosis, after controlling for age, emergency presentation, receipt of resection and comorbidities. The gap was smaller in other stages and sites. For both cancers, patients with emergency presentation persistently had a higher excess hazard of death than those without emergency presentation.

**Conclusion:**

Survival disparities were profound particularly among patients in the stages, which benefit from appropriate and timely treatment. For the patients with emergency presentation, excess hazard of death remained high throughout three years from the diagnosis. Public health measures should be taken to reduce access inequalities to improve survival disparities.

## Introduction

Survival of colorectal cancer (CRC) has improved over the decades. However, cancer survival still varies across countries,^1^ gender and age,^2^ ethnicity^3^ and socioeconomic status (SES).^4–7^ Among them, socioeconomic inequalities in cancer survival are of particular interest as these could be avoided by appropriate public health measurement. In England, despite the healthcare system being based on universal health coverage, CRC has a wide gap in its survival by deprivation: a −7 to −10% difference in 1 year relative survival for CRC between the most affluent and the most deprived groups has been reported.^4^

Considerable effort has been spent on demonstrating the survival gap by hazard ratios (HRs) using the Cox regression; however, it is not easy to interpret the magnitude of the survival gap only from relative measures such as ratios. In particular, for the socioeconomic inequalities, the absolute difference is as important as the relative ratios to understand the magnitude and the direction of the gap properly.^8^

This study aims to present the socioeconomic gap in CRC survival using the flexible parametric model (FPM) developed by Royston and Lambert.^9 10^ We investigated the absolute difference in net survival of colon and rectal cancer, respectively, between the most affluent and the most deprived groups in England. Also, we explore how the excess HRs (EHRs) of the factors associated with survival change over time.

## Methods

### Data sources

Patients diagnosed with primary colon (except appendix) or rectal cancer between January 2010 and March 2013 and followed up until the end of 2014 were included in our analyses. All adult patients (15–99 years) with any stages (I–IV) and histological types were included. A retrospective cohort of the National Cancer Registry data in England, managed by the Office for National Statistics and the Public Health England, were accessed through the Cancer Analysis System.^11^ These data were also linked to several datasets including detailed clinical information, namely, to the National Bowel Cancer Audit data^11 12^ and to the Hospital Episode Statistics (HES) data.^13 14^ Stage information (the fifth edition of the Union for International Cancer Control: UICC TNM Classification^15^) was derived from audit data and the Registry data.^16^ Information on emergency presentation before the first major resection for the primary lesion (ie, emergency presentation recorded at the time of diagnosis or the time of the first open or laparoscopic resection) was established from routes to diagnosis recorded in several health electronic datasets.^17^ Online supplemental material 1 shows the type of surgery identified as major resection.

10.1136/jech-2021-216754.supp1 Supplementary data

Based on a previously established algorithm,^18 19^ comorbidities defined in the Charlson Comorbidity Index,^18 20 21^ and obesity, recorded from 5 to 0 years before the diagnosis of CRC, were extracted from HES data and modified based on its clinical relevance.^22^ These comorbidities were separately defined as chronic or acute; comorbidities that appeared in the HES at least once between 0.5 and 5 years before diagnosis of CRC were characterised as chronic comorbidities. Comorbidities that were recorded for the first time, between the date of diagnosis and 0.5 years before diagnosis, were characterised as acute comorbidities. Details of the comorbidities defined in this study are described in online supplemental material 2.^23^

Based on their residence at the time of cancer diagnosis, we used an ecological measure, income domain of the Index of Multiple Deprivation (2010) quintile to define the deprivation level of patients.^24^

### Outcome

The main outcomes of interest in this study were socioeconomic differences in excess hazard of death and net survival from CRC diagnosis to 3 years after diagnosis.

All outcomes were estimated using survival analysis methods. The entry time for all survival analyses was the date of diagnosis, and hazards of death and survival were derived up to 3 years from diagnosis. The Cox regression analysis was used to estimate the hazard of death in the overall survival setting. The FPM approach was used to estimate excess hazard of death, net survival and to derive differences in these outcomes between the most affluent (SES 1) and the most deprived (SES 5) groups of patients (subtracting excess mortality rate/net survival of the most affluent group from the excess mortality rate/net survival of the most deprived). Net survival is defined as the survival under a hypothetical situation where the cause of death would be solely from a disease of interest, that is, colon or rectal cancer in our study.^25^

The excess hazard of death solely from CRC was derived by comparing the observed overall survival of the patients with CRC with the expected survival of an equivalent population (ie, same sex, age, deprivation group and government regions), using lifetables of the background population (2011, England).^26 27^

### Analysis strategy

Survival analyses were conducted separately for colon and rectal cancer. We started with the Cox regression analysis under the overall survival setting and moved to analysis using FPM under the overall and then the net survival setting. In the first Cox regression analysis, factors potentially associated with survival were explored, and HRs of death by SES were derived.

In the analysis using FPM, in addition to the HRs or EHRs, ‘difference’ measures by SES were explored, after controlling for all potentially associated factors. Graphical measures of the differences in excess hazard of death and net survival between SES 1 and SES 5 were presented for each stage and sex.

Regarding the Cox model, bivariable analysis with the main effect (SES) was conducted for all the other variables one at a time. Each variable with strong evidence for association (p<0.05 in the Wald test) with the outcome was retained in the multivariable model, while age at diagnosis and sex were included as *a priori* confounders. Interaction between SES and stage was added as the primary interest. Some variables, such as stage, tumour grade, emergency presentation and histology were missing in some patients: multiple imputations were conducted for the Cox regression analysis to assess the consistency of the results (online supplemental material 3).^28 29^

Next, we applied the FPM using stpm2. The inclusion of the variables in the FPM was based on the multivariable Cox regression analyses.^9^ Proportional hazard assumption was assessed for each variable using Schoenfeld residuals. The identified variables that breached the proportional hazard assumption were changed to time-varying covariates (TVCs) in the FPM model. When fitting the FPM, the positions of the internal knots for the non-TVCs were set at three points at 90 days, 6 months and 1 year from the date of diagnosis considering a clinically plausible timeline.^30^ For the TVCs, the number of internal knots was reduced from three (baseline hazard) to two: time points at 6 months and 1 year from the date of diagnosis.^9^ After building the FPMs with TVCs for overall survival, the same models were applied to estimate net survival. Stata 14 (StataCorp, College Station, Texas, USA) was used for all analyses.

## Results

A total of 68 169 patients with colon cancer and 38 267 patients with rectal cancer were diagnosed during the study period. For both colon and rectal cancers, crude mortality was around 10% higher in the most deprived group than the most affluent group (online supplemental tables 1 and 2). While the median age for both cancers was over 70 years old, the median age for rectal cancer was 3 years lower than that for colon cancer.

Socioeconomic gradients towards better figures in the most affluent group were noticeable in the proportion of patients with emergency presentation and the number of comorbidities for both colon and rectal cancer. There was no clear socioeconomic trend in stage distribution for colon cancer, but the proportion of patients at stage IV was higher in the most deprived group (20.6%) than the affluent group (17.9%) for rectal cancer. There was no clear socioeconomic gradient in histology and tumour grade, but missingness of tumour grade was higher in the more deprived groups for both cancers. The proportion of the patients who underwent resection was higher in the more affluent groups for both cancers, but the gap in the proportion was relatively smaller for colon cancer.

### Survival analysis with the Cox regression and FPM

The multivariable Cox regression model included patients with 38 070 colon cancer (55.8% of total) and 22 631 rectal cancer (59.1% of total) with complete data. For overall survival, both adjusted HRs of the non-TVCs (tables 1 and 2) and adjusted stage-specific HRs (table 3) showed close agreement between the FPMs and the Cox regression models. There was generally a clear socioeconomic gradient towards higher hazards in the deprived groups for patients with stages I, II and III colon cancer and patients with stages I, II and IV rectal cancer. A similar trend was also confirmed for EHRs in net survival (table 3). The socioeconomic trend was more evident in the Cox regression analyses using imputed data in all stages for both cancers (online supplemental tables 3 and 4).

**Table 1:** HRs and excess HRs of death by multivariable Cox and flexible parametric model for patients with colon cancer, England, January 2010–March 2013

Colon (n=38 070)	Multivariable Cox model		Multivariable FPM with TVCs		Multivariable FPM with TVCs	
	Overall survival		Overall survival		Net survival	
	Adjusted HR*	95% CI	Adjusted HR*	95% CI	Adjusted EHR*	95% CI
SES						
1 (affluent)	Reference		Reference		Reference	
2	1.03	0.97 to 1.08	1.03	0.97 to 1.09	1.01	0.95 to 1.08
3	1.04	0.99 to 1.10	1.04	0.99 to 1.10	1.00	0.94 to 1.07
4	1.13	1.07 to 1.19	1.13	1.07 to 1.19	1.08	1.02 to 1.16
5 (deprived)	1.15	1.09 to 1.22	1.15	1.09 to 1.22	1.08	1.00 to 1.15
Sex			TVC		TVC	
Male	Reference					
Female	0.94	0.91 to 0.98				
Age group			TVC		TVC	
<65	Reference					
65–80	1.43	1.37 to 1.50				
80<	2.32	2.20 to 2.43				
Cancer site†			TVC		TVC	
Right-sided colon	Reference					
Transverse colon	1.01	0.96 to 1.07				
Left-sided colon	0.80	0.76 to 0.83				
Overlapping or unspecified	1.08	0.99 to 1.17				
Stage at diagnosis			TVC		TVC	
I	Reference					
II	1.69	1.36 to 2.11				
III	3.33	2.69 to 4.12				
IV	11.10	9.05 to 13.62				
Tumour grade			TVC		TVC	
G1/G2	Reference					
G3/G4	1.73	1.67 to 1.80				
Emergency presentation			TVC		TVC	
No	Reference					
Yes	1.89	1.82 to 1.96				
Received major resection			TVC		TVC	
Yes	Reference					
No	3.05	2.92 to 3.18				
Number of chronic comorbidities						
0	Reference		Reference		Reference	
1	1.21	1.15 to 1.28	1.21	1.15 to 1.28	1.21	1.13 to 1.30
2+	1.65	1.49 to 1.82	1.64	1.48 to 1.81	1.69	1.49 to 1.91
Number of acute comorbidities			TVC		TVC	
0	Reference					
1	1.23	1.17 to 1.29				
2+	1.66	1.51 to 1.82				

Calculated for complete cases only.

*All variables are mutually adjusted. For SES only, adjusted HRs/EHRs are shown without interaction between SES and stage. For other variables, interaction between SES and stage is adjusted.

†Right-sided colon includes ascending colon, hepatic flexure and caecum. Transverse colon includes transverse colon and splenic flexure. Left-sided colon includes descending colon and sigmoid colon.

EHR, excess hazard ratio; FPM, flexible parametric model; HR, hazard ratio; SES, socioeconomic status; TVC, time-varying covariate.

**Table 2:** HRs and excess HRs of death by multivariable Cox and flexible parametric model for patients with rectal cancer, England, January 2010–March 2013

Rectum (n=22 631)	Multivariable Cox model		Multivariable FPM with TVCs		Multivariable FPM with TVCs	
	Overall survival		Overall survival		Net survival	
	Adjusted HR*	95% CI	Adjusted HR*	95% CI	Adjusted EHR*	95% CI
SES						
1 (affluent)	Reference		Reference		Reference	
2	0.97	0.90 to 1.04	0.97	0.90 to 1.04	0.95	0.87 to 1.04
3	1.05	0.97 to 1.13	1.05	0.97 to 1.13	1.02	0.93 to 1.12
4	1.04	0.96 to 1.12	1.04	0.97 to 1.12	1.00	0.91 to 1.09
5 (deprived)	1.17	1.08 to 1.27	1.17	1.08 to 1.27	1.11	1.01 to 1.22
Sex						
Male	Reference		Reference		Reference	
Female	0.91	0.87 to 0.96	0.91	0.87 to 0.96	0.97	0.91 to 1.03
Age group			TVC		TVC	
<65	Reference					
65–80	1.60	1.51 to 1.70				
80<	2.97	2.78 to 3.17				
Year of diagnosis						
2010	Reference		Reference		Reference	
2011	0.97	0.91 to 1.03	0.96	0.91 to 1.03	0.99	0.92 to 1.07
2012	0.89	0.84 to 0.95	0.89	0.84 to 0.95	0.91	0.85 to 0.98
2013	0.93	0.83 to 1.03	0.94	0.84 to 1.04	1.01	0.89 to 1.14
Cancer site			TVC		TVC	
Rectosigmoid junction	Reference					
Rectum	0.89	0.84 to 0.95				
Overlapping or unspecified	0.70	0.47 to 1.04				
Stage at diagnosis						
I	Reference		Reference		Reference	
II	2.37	1.85 to 3.02	2.38	1.87 to 3.04	6.13	2.98 to 12.62
III	3.26	2.61 to 4.07	3.29	2.64 to 4.11	10.02	4.98 to 20.15
IV	10.10	8.18 to 12.47	10.13	8.20 to 12.51	35.80	17.94 to 71.43
Tumour grade			TVC		TVC	
G1/G2	Reference					
G3/G4	1.80	1.70 to 1.91				
Emergency presentation			TVC		TVC	
No	Reference					
Yes	1.81	1.70 to 1.93				
Received major resection						
Yes	Reference		Reference		Reference	
No	2.89	2.73 to 3.05	2.88	2.72 to 3.04	3.62	3.37 to 3.89
Number of chronic comorbidities						
0	Reference		Reference		Reference	
1	1.38	1.28 to 1.49	1.37	1.27 to 1.48	1.39	1.26 to 1.53
2+	1.99	1.72 to 2.31	1.96	1.70 to 2.27	2.14	1.79 to 2.55
Number of acute comorbidities						
0	Reference		Reference		Reference	
1	1.32	1.22 to 1.42	1.31	1.21 to 1.41	1.36	1.23 to 1.49
2+	1.75	1.50 to 2.05	1.75	1.50 to 2.05	1.88	1.56 to 2.27

Calculated for complete cases only.

*All variables are mutually adjusted. For SES only, adjusted HRs/EHRs are shown without interaction between SES and stage. For other variables, interaction between SES and stage is adjusted.

EHR, excess hazard ratio; FPM, flexible parametric model; HR, hazard ratio; NA, not applicable (not included in multivariable model); SES, socioeconomic status; TVC, time-varying covariate.

**Table 3:** Adjusted stage-specific HRs and excess HRs of death for patients with colon and rectal cancer, England, January 2010–March 2013

	Colon (n=38 070)						Rectum (n=22 631)					
	Overall survival		Overall survival		Net survival		Overall survival		Overall survival		Net survival	
	Cox model^a^		FPM with TVCs^b^		FPM with TVCs^c^		Cox model^d^		FPM with TVCs^e^		FPM with TVCs^f^	
	Adjusted HR	95% CI	Adjusted HR	95% CI	Adjusted EHR	95% CI	Adjusted HR	95% CI	Adjusted HR	95% CI	Adjusted EHR	95% CI
Stage I												
SES 1 (affluent)	Reference		Reference		Reference		Reference		Reference		Reference	
2	1.04	0.79 to 1.37	1.03	0.78 to 1.37	1.10	0.49 to 2.46	1.02	0.77 to 1.34	1.02	0.77 to 1.34	1.33	0.56 to 3.14
3	0.99	0.74 to 1.32	0.99	0.74 to 1.32	0.65	0.23 to 1.82	1.11	0.85 to 1.45	1.11	0.85 to 1.46	1.40	0.59 to 3.29
4	1.07	0.80 to 1.43	1.07	0.80 to 1.43	1.16	0.51 to 2.67	1.14	0.87 to 1.49	1.14	0.87 to 1.50	1.32	0.55 to 3.18
5 (deprived)	1.18	0.88 to 1.59	1.20	0.89 to 1.60	1.56	0.71 to 3.44	1.44	1.09 to 1.90	1.45	1.10 to 1.91	2.00	0.86 to 4.66
Stage II												
SES 1	Reference		Reference		Reference		Reference		Reference		Reference	
2	1.03	0.89 to 1.18	1.02	0.89 to 1.18	0.97	0.76 to 1.25	0.98	0.80 to 1.20	0.98	0.80 to 1.21	0.89	0.64 to 1.24
3	1.11	0.97 to 1.28	1.11	0.97 to 1.28	1.01	0.78 to 1.30	1.15	0.95 to 1.40	1.15	0.95 to 1.40	1.15	0.85 to 1.55
4	1.38	1.20 to 1.58	1.37	1.19 to 1.57	1.53	1.21 to 1.93	1.14	0.94 to 1.39	1.14	0.94 to 1.39	1.16	0.86 to 1.57
5	1.45	1.26 to 1.67	1.43	1.24 to 1.65	1.56	1.22 to 1.99	1.33	1.08 to 1.63	1.33	1.09 to 1.64	1.41	1.04 to 1.91
Stage III												
SES 1	Reference		Reference		Reference		Reference		Reference		Reference	
2	1.16	1.05 to 1.29	1.17	1.05 to 1.29	1.16	1.02 to 1.32	0.98	0.85 to 1.13	0.98	0.84 to 1.13	0.96	0.80 to 1.16
3	1.11	0.99 to 1.23	1.11	1.00 to 1.23	1.06	0.93 to 1.22	1.07	0.93 to 1.24	1.07	0.93 to 1.24	1.04	0.86 to 1.25
4	1.19	1.07 to 1.32	1.20	1.08 to 1.33	1.13	0.98 to 1.29	1.11	0.96 to 1.28	1.11	0.96 to 1.28	1.10	0.91 to 1.32
5	1.33	1.19 to 1.48	1.33	1.19 to 1.49	1.29	1.12 to 1.48	1.08	0.93 to 1.25	1.08	0.93 to 1.25	1.04	0.85 to 1.26
Stage IV												
SES 1	Reference		Reference		Reference		Reference		Reference		Reference	
2	0.96	0.90 to 1.04	0.97	0.90 to 1.04	0.96	0.89 to 1.04	0.95	0.85 to 1.05	0.95	0.85 to 1.05	0.95	0.85 to 1.06
3	1.00	0.93 to 1.07	1.00	0.93 to 1.07	0.98	0.91 to 1.06	1.00	0.90 to 1.11	1.00	0.90 to 1.11	0.99	0.89 to 1.11
4	1.04	0.96 to 1.12	1.04	0.97 to 1.12	1.03	0.95 to 1.11	0.96	0.87 to 1.07	0.97	0.87 to 1.08	0.94	0.84 to 1.05
5	0.99	0.92 to 1.08	0.99	0.92 to 1.08	0.96	0.89 to 1.05	1.15	1.03 to 1.28	1.14	1.03 to 1.27	1.09	0.97 to 1.22

Interaction between SES and stage is added for all models. Calculated for complete cases only.

Model a, b, c: adjusted for sex, age, site, tumour grade, emergency presentation, receipt of major resection, number of chronic and acute comorbidities.

Model d, e, f: adjusted for sex, age, year of diagnosis, site, tumour grade, emergency presentation, receipt of major resection, number of chronic and acute comorbidities.

EHR, excess HR; FPM, flexible parametric model; SES, socioeconomic status; TVC, time-varying covariate.

For the TVCs, HRs and EHRs changed over time (online supplemental tables 5 and 6). For both cancers, the effect of age on hazard of death decreased over time. The effect of emergency presentation on the hazard of death tended to increase over time, especially for colon cancer. The hazard/excess hazard of death for rectal cancer increased over time but was consistently lower than that of rectosigmoid cancer. When comparing the HRs and EHRs of the stages (online supplemental table 5 for colon and table 2 for rectal cancer), EHRs increased substantially in stage IV, that is, the reference group of patients with stage I rarely died from cancer. The effects of major resection and acute comorbidities on EHRs only changed over time among patients with colon cancer, but both were highest in the first period (90 days from diagnosis) (online supplemental table 5).

### Difference in excess mortality rates and net survival between the most affluent and the most deprived groups

**Figure 1:**
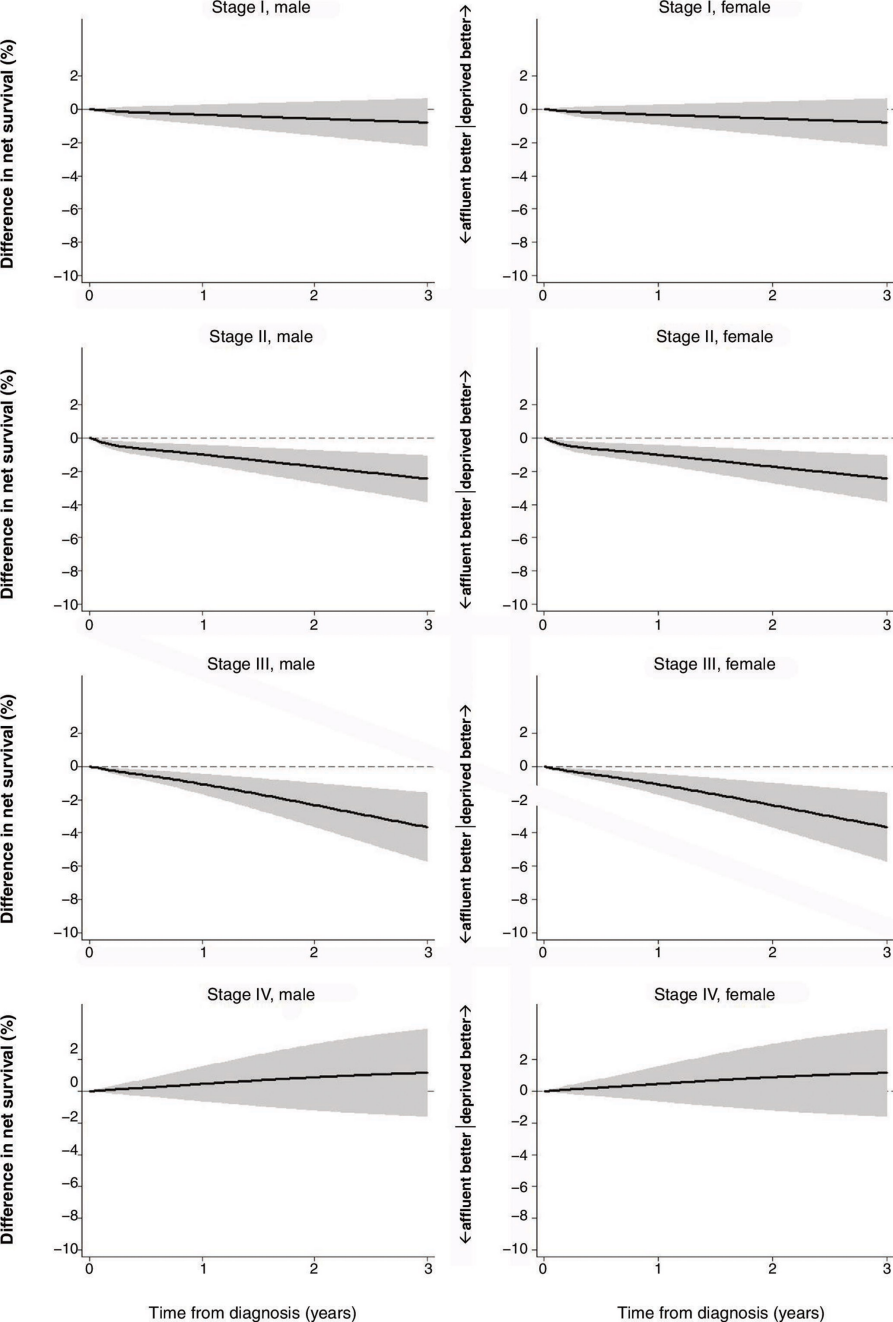
Difference in net survival between SES 1 (the most affluent) and SES 5 (the most deprived), colon cancer, England, January 2010–March 2013. Age group was set at under 65 years old, cancer site at right-sided colon, tumour grade at G1/G2, no emergency presentation, received major resection and no chronic or acute comorbidities. SES, socioeconomic status.

**Figure 2:**
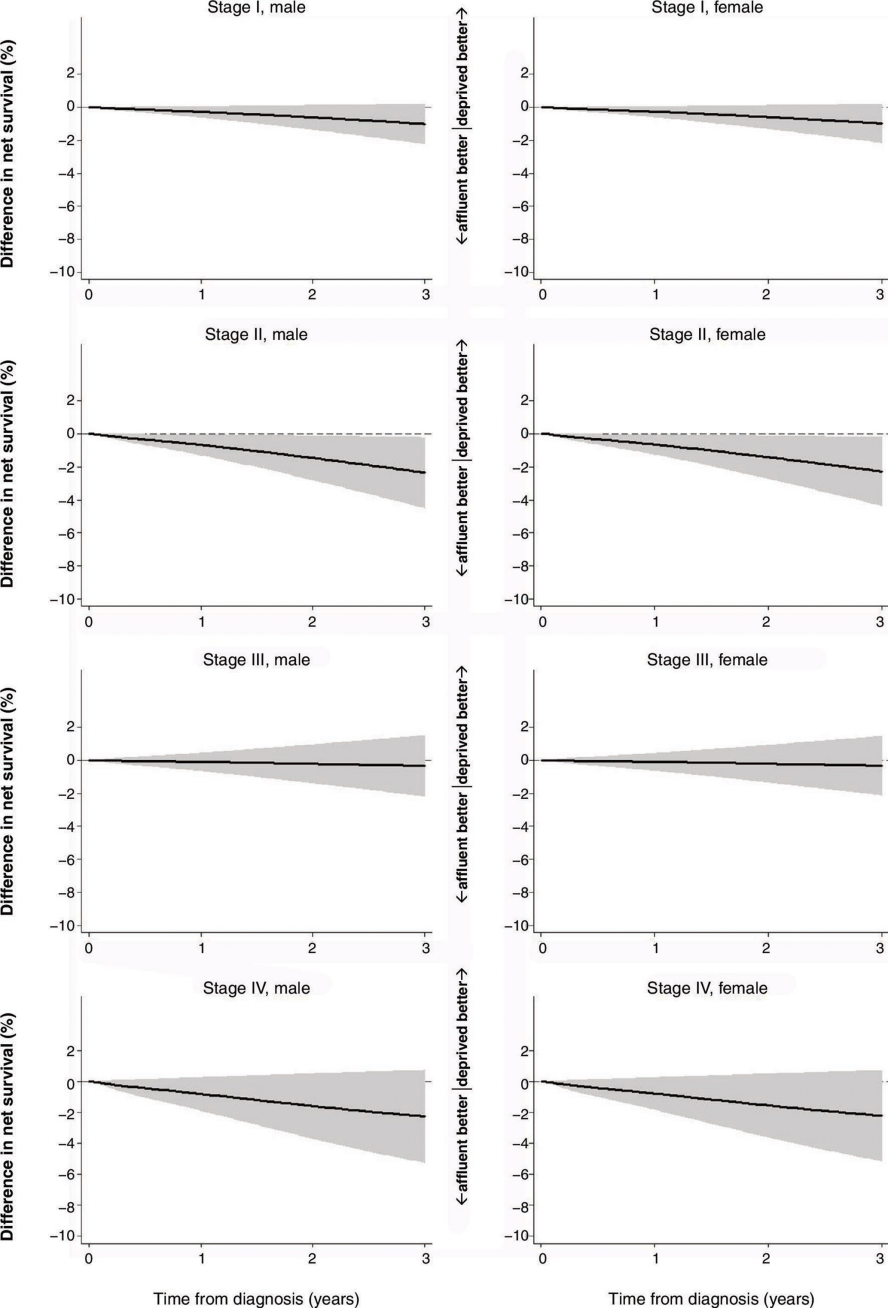
Difference in net survival between SES 1 (the most affluent) and SES 5 (the most deprived), rectal cancer, England, January 2010–March 2013. Age group was set at under 65 years old, cancer site at rectosigmoid junction, tumour grade at G1/G2, no emergency presentation, received major resection and no chronic or acute comorbidities. Year of diagnosis was set at 2010. SES, socioeconomic status.

Based on the final FPM models, two measures of difference were graphically estimated: difference in excess mortality rates (ie, excess hazard of death) between the most affluent and the most deprived groups (online supplemental figures 1 and 2) and difference in net survival between the two groups (figures 1 and 2).

For colon cancer, the most deprived group had a larger excess mortality rate than the most affluent group in stages I, II and III. In stages II and III, the difference hits a sharp peak around 20 per 1000 person-years at the very beginning and then declined, but in stage III, the figure again showed a gradual increase over time (online supplemental figure 1). As expected from the difference in excess hazard of death, the most affluent group had higher net survival than the most deprived group in stages I, II and III (online supplemental figure 3). The difference in net survival was largest in stage III, reaching approximately −4% (−3.7%, 95% CI −1.6 to −5.7% in men, −3.6%, 95% CI −1.6 to −5.7% in women) at the 3-year point (figure 1).

For rectal cancer, in stages I and III, the gap in excess hazard was almost null, whereas in stages II and IV, a difference of more than 5/1,000 person-years was observed (online supplemental figure 2). The most deprived group had poorer net survival than the most affluent group in all stages (online supplemental figure 4); however, the adjusted difference did not expand more than −3% for all stages. Only in stage II, the 95% CI of the gap remained below 0 throughout (−2.3%, 95% CI −0.2 to −4.5% in men, −2.3%, 95% CI −0.2 to −4.3% in women) (figure 2).

## Discussion

We estimated the difference in EHRs and net survival between the most affluent and the most deprived groups using FPM for patients diagnosed with CRC in England during 2010–2013.

Our results revealed that the socioeconomic gap in net survival was noticeable among patients with stages II and III colon cancer and patients with stage II rectal cancer even after controlling for associated factors. Patients at these stages can potentially be cured if they receive appropriate and timely treatment.

The resection was provided relatively equally for all SES groups for colon cancer, whereas it was not equally distributed for rectal cancer (online supplemental tables 1 and 2). Considering that a survival gap existed in colon cancer, quality of care (both surgical and postoperative care) might have differed by SES, possibly across the hospitals where they were treated. For rectal cancer, patients might be selected for resection based on unmeasured factors. However, the socioeconomic gaps in net survival were relatively small for rectal cancer. These results together imply that the existing inequalities in receipt of major resection may not play a major role in the survival inequalities for rectal cancer. Other unmeasured factors, such as receipt of neoadjuvant therapy, access issues or behavioural factors, may be more important for the survival inequalities observed in patients with stage II rectal cancer.

Increased age, no resection and increased number of acute comorbidities affected survival, especially shortly after diagnosis for colon cancer. The decreasing trend in hazard of death for aged patients over time suggests that frail patients died mostly soon after diagnosis. Emergency presentation affects survival increasingly over time for both cancers, suggesting that the patients with emergency presentations should be followed up carefully for a long period.

Among the patients with colon cancer, adjusted excess hazard of death for the patients with transverse colon cancer was higher than that of those with right-sided colon cancer during the early period following diagnosis (online supplemental table 5). One of the potential explanations could be a higher risk of postoperative complications (eg, leaks) due to the anatomical structure of the blood supply to the transverse colon.^31^ Also, our study showed that the hazard of death for the patients with left-sided colon cancer was consistently lower than that of right-sided colon cancer, which agrees with some other studies.^32 33^ Although we adjusted for some known explanations for the difference in survival such as age and stage,^34^ we found a lower survival for right-sided colon cancer. The survival difference could be associated with some unmeasured confounders, such as biological factors. Survival of cancer with *BRAF* mutations, often associated with right-sided colon cancer, may have resulted in poorer survival than cancer with *KRAS* mutations, often seen in left-sided colon cancer.^35–37^ The increased mortality rate for rectal cancer after 6 months (online supplemental table 6) may suggest the local recurrence, which is common in rectal cancer compared with cancer of the rectosigmoid junction.

Finally, excess mortality showed a three to four-fold increase in patients without resection compared with patients with resection for both cancers. Careful interpretation is needed for the high hazard of death in those without resection. Patients without resection may have died not because they were not given the opportunity to have the resection but because they did not live long enough for it to take place; reverse causality may exist.

Regarding the statistical methods used in this study, the advantages of using the FPM are that the FPM allows us to estimate the baseline survival function and also enables us to illustrate the absolute ‘difference’ smoothly in hazard and survival over time.^9^ Another advantage of using the FPM is that it enables us to easily deal with time-varying effects when proportional hazard assumption does not hold in the Cox regression model. By comparing the results of HR/EHRs derived by the Cox model, FPM for overall survival and FPM for net survival (tables 1 and 2), we revealed that the assumption of the proportional hazard did not hold in some key factors (eg, stage for colon cancer and emergency presentation in both cancers). The effect of stage and comorbidities on survival was substantial for net survival compared with that for overall survival.

From a clinical and public health perspectives, measuring absolute difference as well as measuring relative ratios is useful to understand the magnitude of socioeconomic inequalities.^38^ Relative ratios may look remarkable even when the absolute difference is insignificant.^39^ Our study on both HR/EHRs and the difference in net survival by stage provides transparency on reporting, an unbiased estimate to interpret the magnitude of the socioeconomic inequalities and which point of care the policymakers should focus and improve.

Our study has limitations. The HRs, EHRs and net survival derived by FPM can only be estimated for complete data because FPM does not currently support multiply imputed data. For both cancers, less than 60% of the total patients were analysed. However, the primary issue here is missing data, not the fact that the imputed data cannot be used. The main results are derived from two complete-case analyses using Cox regression and FPM. We also performed Cox regression after multiple imputations. We have not applied the excess hazard model (using FPM) on the imputed data because of methodological limitations, that is, the lack of compatibility between the imputation model and the substantive model (FPM). However, all these analyses (table 3 and online supplemental table 4) imply that the results from the FPM are likely to be underestimated but less likely to be overestimated.

Severity of comorbidities was lacking in the data and misclassification may exist. However, we complemented this lack of severity by differentiating acute and chronic comorbidities. It is important that we further investigate the ways in which chronicity and severity of the comorbidities affect socioeconomic inequalities in cancer care and survival.

In conclusion, our study found that socioeconomic gaps in CRC survival varied by colon or rectum and also by stage. Survival disparities remained over time in stages II and III for colon and stage II for rectal cancer even after controlling for age, emergency presentation, receipt of resection and comorbidities. Emergency presentations affect survival not only at the initial point of care but also afterwards for a longer period. Other access, for example, triage of the emergency cases, quality of postoperative care for colon cancer (eg, complication rates, failure-to-rescue rates) and receipt of neoadjuvant therapy for rectal cancer might have differed by SES. For the emergency cases, efforts should be made, first, to triage the vital emergency cases (eg, with perforation). Patients with obstruction could be transferred to specialised department for later surgery by colorectal surgeon. Nonvital emergency patients should be referred to the 2-week referral pathway. Also, if postoperative complications happen, colorectal surgeons should be called for monitoring the complications, rather than nonspecialised surgeons. These management systems may differ across hospitals and may lead to differences in survival by SES. Future research should investigate disparities in those unmeasured factors. Public health measures should be taken to evaluate and reduce these potential access inequalities to fill the survival gap.

**What is already known on this subject**

- Socioeconomic inequalities in colorectal cancer survival have been persistently reported in England.
- Socioeconomic inequalities in cancer survival are often summarised by relative measures such as hazard ratios, while absolute difference is as informative as the ratios and directly interpretable from the public health perspective.

**What this study adds**

- Absolute gap in net survival between the most and the least deprived groups reached approximately −4% at 3 years from colorectal cancer diagnosis at maximum, after controlling for age, emergency presentation, comorbidities and receipt of surgery.
- Excess hazard of death for the patients with emergency presentation persisted or even increased over time. Patients with emergency presentation should be carefully followed up even after 1 year since the diagnosis of colorectal cancer.
- Evaluation of the management of emergency cases and postoperative care is needed for assessing disparities in cancer care and socioeconomic gaps in colorectal cancer survival.

## Data Availability

Data may be obtained from a third party and are not publicly available. Data were provided by Public Health England (PHE). Our data sharing agreement with PHE clearly stipulates that they cannot be shared with any third party without the prior written consent of PHE.
