# Supplementary material for: Socioeconomic gaps over time in colorectal cancer survival in England: flexible parametric survival analysis
Source: J Epidemiol Community Health. 2021 May 28;75(12):1155–64. doi: 10.1136/jech-2021-216754 (PMC8588290; doi:10.1136/jech-2021-216754)
Supplement: Supplementary data [file jech-2021-216754supp001.pdf]

Title: Socioeconomic Gaps Over Time in Colorectal Cancer Survival in England: Flexible Parametric Survival Analysis

Mari Kajiwara Saito[1*,2], Manuela Quaresma[1], Helen Fowler[3], Sara Benitez Majano[1], Bernard Rachet[1]

1. Inequalities in Cancer Outcomes Network, Department of Non-communicable Disease Epidemiology, Faculty of Epidemiology and Population Health, London School of Hygiene & Tropical Medicine, Keppel Street, London, WC1E 7HT, United Kingdom

2. IMS Tokyo Katsushika General Hospital, 4-18-1, Nishi-Shin-Koiwa, Katsushika-ku, Tokyo, 124-0025, Japan

3. Department of Non-communicable Disease Epidemiology, Faculty of Epidemiology and Population Health, London School of Hygiene & Tropical Medicine, Keppel Street, London, WC1E 7HT, United Kingdom

* mari.kajiwara@lshtm.ac.uk

Kajiwara Saito M, *et al. J Epidemiol Community Health* 2021; 75:1155–1164. doi: 10.1136/jech-2021-216754

Supplementary Material 1. Operation Code and Name for Colon and Rectal Cancer.

Regarding the extraction of the date and type of operation procedure of the first major surgery, we defined the NBOCA data as the priority.

Information on operation procedure and date of the first major surgery was extracted from HES if NBOCA data had no information.

A major operation for the primary lesion was extracted from 30 days before diagnosis to 180 days after diagnosis.

NBOCA data covered 82.0% and 80.3% of the total information on the first major surgery for the primary lesion in colon and rectal cancers, respectively.

Continued from Supplementary Material 1.

Code	Operation Name for Colon Cancer
H04.1	Proctocolectomy NEC, Panproctocolectomy and Ileostomy
H04.2	Panproctocolectomy and anastomosis of ileum to anus and creation of pouch HFQ
H04.3	Panproctocolectomy and anastomosis of ileum to anus NEC
H04.8	Other specified total excision of colon and rectum
H04.9	Panproctocolectomy NEC, Total excision of colon and rectum, unspecified-
H05.1	Total colectomy and anastomosis of ileum to rectum
H05.2	Total colectomy and ileostomy and creation of rectal fistula HFQ
H05.3	Total colectomy and ileostomy NEC
H05.8	Total excision of colon, other specified
H05.9	Total excision of colon, Unspecified
H06.1	Extended right hemicolectomy and end to end anastomosis
H06.2	Extended right hemicolectomy and anastomosis of ileum to colon
H06.3	Extended right hemicolectomy and anastomosis NEC
H06.4	Extended right hemicolectomy and ileostomy HFQ
H06.8	Other specified extended excision of right hemicolon
H06.9	Extended excision of Right hemicolon, unspecified, excision of Right colon and surrounding tissue
H07.1	Right hemicolectomy and end to end anastomosis of ileum to colon, Ileocaecal resection
H07.2	Right hemicolectomy and side to side anastomosis of ileum to transverse colon,
H07.3	Right hemicolectomy and anastomosis NEC
H07.4	Right hemicolectomy and ileostomy HFQ
H07.8	Other specified other excision of right hemicolon
H07.9	Other excision of right hemicolon, unspecified; Right hemicolectomy NEC
H08.1	Transverse colectomy and end to end anastomosis
H08.2	Transverse colectomy and anastomosis of ileum to colon
H08.3	Transverse colectomy and anastomosis NEC
H08.4	Transverse colectomy and ileostomy HFQ
H08.5	Transverse colectomy and exteriorisation of bowel NEC CODE COLOSTOMY SPERATELY
H08.8	Other specified excision of transverse colon
H08.9	Excision of transverse colon, unspecified
H09.1	Left hemicolectomy and end to end anastomosis of colon to rectum
H09.2	Left hemicolectomy and end to end anastomosis of colon to colon
H09.3	Left hemicolectomy and anastomosis NEC
H09.4	Left hemicolectomy and ileostomy HFQ
H09.5	Left hemicolectomy and exteriorisation of bowel NEC CODE COLOSTOMY SEPERATELY
H09.8	Excision of left hemicolon, Other specified
H09.9	Left hemicolectomy NEC, Excision of left hemicolon, Unspecified
H10.1	Sigmoid colectomy and end to end anastomosis of ileum to rectum
H10.2	Sigmoid colectomy and anastomosis of colon to rectum
H10.3	Sigmoid colectomy and anastomosis NEC
H10.4	Sigmoid colectomy and ileostomy HFQ
H10.5	Sigmoid colectomy and exteriorisation of bowel NEC
H10.8	Other specified excision of sigmoid colon
H10.9	Unspecified excision of sigmoid colon
H11.1	Colectomy and end to end anastomosis of colon to colon NEC
H11.2	Colectomy and side to side anastomosis of ileum to colon NEC
H11.3	Colectomy and anastomosis NEC
H11.4	Colectomy and ileostomy NEC
H11.5	Colectomy and exteriorisation of bowel CODE COLOSTOMY SEPERATELY
H11.8	Other excision of colon, other specified
H11.9	Hemicolectomy NEC; Colectomy NEC, Other excision of colon, unspecified;

Continued from Supplementary Material 1.

Code	Operation Name for Colon Cancer
H29.1	Subtotal excision of colon and rectum and creation of colonic pouch and anastomosis of colon to anus
H29.2	Subtotal excision of colon and rectum and creation of colonic pouch NEC
H29.3	Subtotal excision of colon and creation of colonic pouch and anastomosis of colon to rectum
H29.4	Subtotal excision of colon and creation of colonic pouch NEC
H29.8	Subtotal excision of colon, Other specified
H29.9	Subtotal excision of colon, Unspecified
H33.1	Abdominoperineal excision of rectum and end colostomy; APR; SCAPER
H33.2	Proctectomy and anastomosis of colon to anus
H33.3	Anterior resection of rectum and anastomosis of colon to rectum using staples
H33.4	Anterior resection of rectum and anastomosis NEC
H33.5	Hartmann procedure, Rectosigmoidectomy and closure of rectal stump and exteriorisation of bowel CODE COLOSTOMY SEPERATELY
H33.6	Anterior resection of rectum and exteriorisation, CODE COLOSTOMY SEPARATELY
H33.7	Perineal resection of rectum HFQ
H33.8	Anterior Resection of Rectum NEC, Rectosigmoidectomy and anastomosis of colon to rectum Excision of rectum, other specified
H33.9	Rectosigmoidectomy NEC, Excision of rectum, unspecified;
H34.1	Open excision of lesion of rectum: Open removal of polyp; Yorke Mason
H40.1	Trans-sphincteric excision of mucosa of rectum
H40.2	Trans-sphincteric excision of lesion of rectum
H40.8	Other specified operations on rectum through anal sphincter
H40.9	Unspecified operations on rectum through anal sphincter
X14.1	Total exenteration of pelvis
X14.3	Posterior exenteration of pelvis
X14.8	Other specified clearance of pelvis

Kajiwara Saito M, *et al. J Epidemiol Community Health* 2021; 75:1155–1164. doi: 10.1136/jech-2021-216754

Continued from Supplementary Material 1.

Code	Operation Name for Rectal Cancer
H04.1	Proctocolectomy NEC, Panproctocolectomy and Ileostomy
H04.2	Panproctocolectomy and anastomosis of ileum to anus and creation of pouch HFQ
H04.3	Panproctocolectomy and anastomosis of ileum to anus NEC
H04.8	Other specified total excision of colon and rectum
H04.9	Panproctocolectomy NEC, Total excision of colon and rectum, unspecified-
H05.1	Total colectomy and anastomosis of ileum to rectum
H05.2	Total colectomy and ileostomy and creation of rectal fistula HFQ
H05.3	Total colectomy and ileostomy NEC
H05.8	Total excision of colon, other specified
H05.9	Total excision of colon, Unspecified
H06.1	Extended right hemicolectomy and end to end anastomosis
H06.2	Extended right hemicolectomy and anastomosis of ileum to colon
H06.3	Extended right hemicolectomy and anastomosis NEC
H06.4	Extended right hemicolectomy and ileostomy HFQ
H06.9	Extended excision of Right hemicolon, unspecified, excision of Right colon and surrounding tissue
H07.1	Right hemicolectomy and end to end anastomosis of ileum to colon, Ileocaecal resection
H07.2	Right hemicolectomy and side to side anastomosis of ileum to transverse colon,
H07.3	Right hemicolectomy and anastomosis NEC
H07.4	Right hemicolectomy and ileostomy HFQ
H07.8	Other specified other excision of right hemicolon
H07.9	Other excision of right hemicolon, unspecified; Right hemicolectomy NEC
H08.1	Transverse colectomy and end to end anastomosis
H08.3	Transverse colectomy and anastomosis NEC
H08.4	Transverse colectomy and ileostomy HFQ
H08.5	Transverse colectomy and exteriorisation of bowel NEC CODE COLOSTOMY SPERATELY
H08.8	Other specified excision of transverse colon
H09.1	Left hemicolectomy and end to end anastomosis of colon to rectum
H09.2	Left hemicolectomy and end to end anastomosis of colon to colon
H09.3	Left hemicolectomy and anastomosis NEC
H09.4	Left hemicolectomy and ileostomy HFQ
H09.5	Left hemicolectomy and exteriorisation of bowel NEC CODE COLOSTOMY SEPERATELY
H09.8	Excision of left hemicolon, Other specified
H09.9	Left hemicolectomy NEC, Excision of left hemicolon, Unspecified
H10.1	Sigmoid colectomy and end to end anastomosis of ileum to rectum
H10.2	Sigmoid colectomy and anastomosis of colon to rectum
H10.3	Sigmoid colectomy and anastomosis NEC
H10.4	Sigmoid colectomy and ileostomy HFQ
H10.5	Sigmoid colectomy and exteriorisation of bowel NEC
H10.8	Other specified excision of sigmoid colon
H10.9	Unspecified excision of sigmoid colon
H11.1	Colectomy and end to end anastomosis of colon to colon NEC
H11.2	Colectomy and side to side anastomosis of ileum to colon NEC
H11.3	Colectomy and anastomosis NEC
H11.4	Colectomy and ileostomy NEC
H11.5	Colectomy and exteriorisation of bowel CODE COLOSTOMY SEPERATELY
H11.8	Other excision of colon, other specified
H11.9	Hemicolectomy NEC; Colectomy NEC, Other excision of colon, unspecified;

Kajiwara Saito M, *et al. J Epidemiol Community Health* 2021; 75:1155–1164. doi: 10.1136/jech-2021-216754

Continued from Supplementary Material 1.

Code	Operation Name for Rectal Cancer
H29.1	Subtotal excision of colon and rectum and creation of colonic pouch and anastomosis of colon to anus
H29.2	Subtotal excision of colon and rectum and creation of colonic pouch NEC
H29.3	Subtotal excision of colon and creation of colonic pouch and anastomosis of colon to rectum
H29.4	Subtotal excision of colon and creation of colonic pouch NEC
H29.8	Subtotal excision of colon, Other specified
H29.9	Subtotal excision of colon, Unspecified
H33.1	Abdominoperineal excision of rectum and end colostomy; APR; SCAPER
H33.2	Proctectomy and anastomosis of colon to anus
H33.3	Anterior resection of rectum and anastomosis of colon to rectum using staples
H33.4	Anterior resection of rectum and anastomosis NEC
H33.5	Hartmann procedure, Rectosigmoidectomy and closure of rectal stump and exteriorisation of bowel CODE COLOSTOMY SEPERATELY
H33.6	Anterior resection of rectum and exteriorisation, CODE COLOSTOMY SEPARATELY
H33.7	Perineal resection of rectum HFQ
H33.8	Anterior Resection of Rectum NEC, Rectosigmoidectomy and anastomosis of colon to rectum Excision of rectum, other specified
H33.9	Rectosigmoidectomy NEC, Excision of rectum, unspecified;
H34.1	Open excision of lesion of rectum: Open removal of polyp; Yorke Mason
H34.2	Open cauterisation of lesion of rectum, Diathermy
H34.5	Open destruction of lesion of rectum NEC
H34.8	Open removal of lesion of rectum, other specified
H40.1	Trans-sphincteric excision of mucosa of rectum
H40.2	Trans-sphincteric excision of lesion of rectum
H40.3	Trans-sphincteric destruction of lesion of rectum
H40.8	Other specified operations on rectum through anal sphincter
H40.9	Unspecified operations on rectum through anal sphincter
X14.1	Total exenteration of pelvis
X14.2	Anterior exenteration of pelvis
X14.3	Posterior exenteration of pelvis
X14.8	Other specified clearance of pelvis
X14.9	Clearance of pelvis, unspecified

Supplementary Material 2. Definition of Chronic and Acute Comorbidities.

Ten and 14 comorbidities of the 17 comorbidities in the Charlson Comorbidity Index were selected for chronic and acute comorbidities, respectively, on the basis of their clinical relevance to the selection and timeliness of treatment for CRC e.g. invasive or less invasive treatment, curative or palliative treatment.

Obesity defined here as body mass index: BMI > 30 was included independently in the acute phase only 0 to 0.5 years before CRC diagnosis because BMI is a reversible condition i.e. a time-varying variable and may confound with stage at diagnosis.

Unlike the Charlson Comorbidity Index, the comorbidities in our study were counted but not assigned a weight.

Chronic Comorbidities	Count	Acute Comorbidities	Count
Chronic heart failure	1	Chronic heart failure	1
Dementia	1	Dementia	1
Chronic pulmonary disease	1	Chronic pulmonary disease	1
Connective tissue disease	1	Connective tissue disease	1
Diabetes mellitus with end organ complication	1	Diabetes mellitus with end organ complication	1
Hemiplegia	1	Hemiplegia	1
Chronic renal disease, moderate to severe	1	Chronic renal disease, moderate to severe	1
Liver disease, moderate to severe	1	Liver disease, moderate to severe	1
HIV Human Immunodeficiency Virus infection	1	HIV Human Immunodeficiency Virus infection	1
Malignancy not colorectal cancer	1	Myocardial infarction	1
		Peripheral vascular disease	1
		Cerebrovascular disease	1
		Peptic ulcer disease	1
		Malignancy not colorectal cancer	1

Kajiwara Saito M, *et al. J Epidemiol Community Health* 2021; 75:1155–1164. doi: 10.1136/jech-2021-216754

Supplementary Material 3. Description of Methods for Multiple Imputation Used for Sensitivity Analysis.

	Colon Cancer					Rectal Cancer				
	SES					SES				
	1	2	3	4	5	1	2	3	4	5
**Stage (%)**										
I	13.4	12.5	12.7	11.7	11.7	23.9	22.3	22.6	21.0	19.9
II	27.2	27.6	26.6	26.2	26.3	20.7	20.7	20.3	21.0	20.0
III	25.7	25.7	25.2	25.0	25.2	29.6	29.9	29.8	29.2	29.8
IV	33.6	34.2	35.5	37.0	36.7	25.9	27.1	27.3	28.8	30.4
**Histology (%)**										
Adenocarcinoma	99.2	99.1	99.1	99.2	99.1	97.5	97.8	97.2	97.1	96.4
Non-adenocarcinoma	0.8	0.9	0.9	0.8	0.9	2.5	2.2	2.8	2.9	3.6
**Tumour grade (%)**										
G1/G2	79.3	79.5	78.6	79.6	79.5	86.5	86.9	85.8	86.0	86.1
G3/G4	20.7	20.5	21.4	20.4	20.5	13.5	13.1	14.2	14.0	13.9
**Emergency presentation (%)**										
No	76.3	74.6	73.6	71.1	67.8	90.0	89.0	87.8	86.2	82.9
Yes	23.7	25.4	26.4	28.9	32.2	10.0	11.0	12.2	13.8	17.1

Abbreviations: SES, socioeconomic status.

Four variables, namely, stage at diagnosis, histology, tumour grade and emergency presentation were missing in some patients.

The missingness was highest in stage with 30.6% and 27.3% for colon and rectal cancer, respectively.

Tumour grade was missing at 24.2% and 22.3%, emergency presentation at 9.9% and 6.7%, and histology at less than 3% for colon and rectal cancer, respectively.

Those variables were multiply imputed for 30 times under the missing at random assumption.

Following variables were used for the multiple imputation: sex, age group, cancer site, number of chronic and acute comorbidities, receipt of major surgery, vital status, Nelson-Aalen estimator and government office region.

The distributions of stage, histology, tumour grade and emergency presentation after the imputation were as follows.

When evaluating association between each variable and survival in the Cox regression, the Wald test, instead of likelihood ratio test, was used for both imputed and completed data to account for the uncertainty in imputed data.

For FPM, complete data only were used because imputed data are not technically supported to use in FPM.

Therefore, analyses using imputed data in the first analysis Cox regression were considered sensitivity analyses and results are shown in Appendix Table 3.

Kajiwara Saito M, *et al. J Epidemiol Community Health* 2021; 75:1155–1164. doi: 10.1136/jech-2021-216754

Supplementary Table 1. Baseline Characteristics of Patients with Colon Cancer, England, January 2010–March 2013.

			SES									
	**Total**		**1 (affluent)**		**2**		**3**		**4**		**5 (deprived)**	
	**No.**	**%**	**No.**	**%**	**No.**	**%**	**No.**	**%**	**No.**	**%**	**No.**	**%**
Total number	68,169	100.0	14,917	21.9	15,112	22.2	14,339	21.0	13,415	19.7	10,386	15.2
Death at the end of follow up	31,766	46.6	6,315	42.3	6,798	45.0	6,685	46.6	6,651	49.6	5,317	51.2
Median age at diagnosis	74.1		73.7		74.3		74.3		74.3		73.4	
IQR	65.3 to 81.5		65.3 to 81.0		65.6 to 81.7		65.7 to 81.8		65.2 to 81.7		64.1 to 81.1	
Female	32,150	47.2	6,875	46.1	7,038	46.6	6,843	47.7	6,472	48.2	4,922	47.4
Year of diagnosis												
2010	20,672	30.3	4,409	29.6	4,643	30.7	4,449	31.0	4,027	30.0	3,144	30.3
2011	21,191	31.1	4,699	31.5	4,687	31.0	4,456	31.1	4,177	31.1	3,172	30.5
2012	21,213	31.1	4,647	31.2	4,670	30.9	4,383	30.6	4,198	31.3	3,315	31.9
2013	5,093	7.5	1,162	7.8	1,112	7.4	1,051	7.3	1,013	7.6	755	7.3
Cancer site[a]												
Right-sided colon	27,616	40.5	6,104	40.9	6,054	40.1	5,824	40.6	5,445	40.6	4,189	40.3
Transverse colon	7,984	11.7	1,752	11.7	1,776	11.8	1,707	11.9	1,590	11.9	1,159	11.2
Left-sided colon	26,887	39.4	5,923	39.7	5,995	39.7	5,679	39.6	5,246	39.1	4,044	38.9
Overlapping or unspecified	5,682	8.3	1,138	7.6	1,287	8.5	1,129	7.9	1,134	8.5	994	9.6
Stage at diagnosis												
I	5,812	8.5	1,359	9.1	1,274	8.4	1,242	8.7	1,089	8.1	848	8.2
II	13,440	19.7	2,979	20.0	3,041	20.1	2,789	19.5	2,577	19.2	2,054	19.8
III	12,595	18.5	2,793	18.7	2,813	18.6	2,598	18.1	2,442	18.2	1,949	18.8
IV	15,445	22.7	3,270	21.9	3,352	22.2	3,236	22.6	3,186	23.8	2,401	23.1
Missing	20,877	30.6	4,516	30.3	4,632	30.7	4,474	31.2	4,121	30.7	3,134	30.2
Histology												
Adenocarcinoma	66,024	96.9	14,413	96.6	14,635	96.8	13,858	96.7	13,035	97.2	10,083	97.1
Non-adenocarcinoma	565	0.8	116	0.8	128	0.9	120	0.8	105	0.8	96	0.9
Missing	1,580	2.3	388	2.6	349	2.3	361	2.5	275	2.1	207	2.0
Tumour grade												
G1, G2	41,998	61.6	9,472	63.5	9,449	62.5	8,758	61.1	8,100	60.4	6,219	59.9
G3, G4	9,655	14.2	2,221	14.9	2,142	14.2	2,081	14.5	1,811	13.5	1,400	13.5
Missing	16,516	24.2	3,224	21.6	3,521	23.3	3,500	24.4	3,504	26.1	2,767	26.6
Emergency presentation												
No	45,320	66.5	10,289	69.0	10,216	67.6	9,678	67.5	8,705	64.9	6,432	61.9
Yes	16,122	23.7	3,138	21.0	3,368	22.3	3,329	23.2	3,386	25.2	2,901	27.9
Missing	6,727	9.9	1,490	10.0	1,528	10.1	1,332	9.3	1,324	9.9	1,053	10.1
Received major resection												
Yes	45,332	66.5	10,146	68.0	10,197	67.5	9,520	66.4	8,736	65.1	6,733	64.8
No	22,837	33.5	4,771	32.0	4,915	32.5	4,819	33.6	4,679	34.9	3,653	35.2

Continued from Supplementary Table 1.

	SES											
	**Total**		**1 (affluent)**		**2**		**3**		**4**		**5 (deprived)**	
	**No.**	**%**	**No.**	**%**	**No.**	**%**	**No.**	**%**	**No.**	**%**	**No.**	**%**
Number of chronic comorbidities												
0	58,323	85.6	13,155	88.2	13,086	86.6	12,330	86.0	11,292	84.2	8,460	81.5
1	7,849	11.5	1,468	9.8	1,628	10.8	1,597	11.1	1,674	12.5	1,482	14.3
2+	1,993	2.9	292	2.0	398	2.6	412	2.9	447	3.3	444	4.3
Number of acute comorbidities												
0	56,534	82.9	12,767	85.6	12,716	84.2	11,917	83.1	10,925	81.5	8,209	79.0
1	9,565	14.0	1,792	12.0	2,010	13.3	2,013	14.0	2,018	15.1	1,732	16.7
2+	2,066	3.0	356	2.4	386	2.6	409	2.9	470	3.5	445	4.3
Obesity at diagnosis	971	1.4	139	0.9	182	1.2	228	1.6	228	1.7	194	1.9

Abbreviations: G, tumour grade; IQR, interquartile range; NA, not applicable; SES, socioeconomic status. [a] Right-sided colon includes ascending colon, hepatic flexure and caecum. Transverse colon includes transverse colon and splenic flexure. Left-sided colon includes descending colon and sigmoid colon.

Supplementary Table 2. Baseline Characteristics of Patients with Rectal Cancer, England, January 2010–March 2013.

			SES									
	Total		1 (affluent)		2		3		4		5 (deprived)	
	No.	%	No.	%	No.	%	No.	%	No.	%	No.	%
Total number	38,267	100.0	7,977	20.8	8,363	21.9	8,057	21.1	7,649	20.0	6,221	16.3
Death at the end of follow up	15,668	40.9	2,913	36.5	3,205	38.3	3,287	40.8	3,328	43.5	2,935	47.2
Median age at diagnosis	70.8		70.8		70.8		70.9		71.2		70.1	
IQR	62.2, 79.1		62.3, 78.8		62.4, 79.1		62.5, 79.2		62.3, 79.5		60.7, 78.6	
Female	14,238	37.2	2,982	37.4	3,130	37.4	2,967	36.8	2,917	38.1	2,242	36.0
Year of diagnosis												
2010	11,621	30.4	2,417	30.3	2,575	30.8	2,413	30.0	2,299	30.1	1,917	30.8
2011	11,793	30.8	2,475	31.0	2,567	30.7	2,478	30.8	2,344	30.6	1,929	31.0
2012	12,019	31.4	2,504	31.4	2,605	31.2	2,560	31.8	2,457	32.1	1,893	30.4
2013	2,834	7.4	581	7.3	616	7.4	606	7.5	549	7.2	482	7.8
Cancer site												
Rectosigmoid junction	7,247	18.9	1,489	18.7	1,591	19.0	1,489	18.5	1,437	18.8	1,241	20.0
Rectum	30,771	80.4	6,446	80.8	6,733	80.5	6,511	80.8	6,153	80.4	4,928	79.2
Overlapping or unspecified	249	0.7	42	0.5	39	0.5	57	0.7	59	0.8	52	0.8
Stage at diagnosis												
I	6,355	16.6	1,417	17.8	1,408	16.8	1,379	17.1	1,220	16.0	931	15.0
II	5,866	15.3	1,229	15.4	1,300	15.5	1,223	15.2	1,195	15.6	919	14.8
III	8,312	21.7	1,720	21.6	1,842	22.0	1,764	21.9	1,635	21.4	1,351	21.7
IV	7,286	19.0	1,426	17.9	1,566	18.7	1,518	18.8	1,497	19.6	1,279	20.6
Missing	10,448	27.3	2,185	27.4	2,247	26.9	2,173	27.0	2,102	27.5	1,741	28.0
Histology												
Adenocarcinoma	36,240	94.7	7,581	95.0	7,956	95.1	7,621	94.6	7,229	94.5	5,853	94.1
Non-adenocarcinoma	1,025	2.7	192	2.4	182	2.2	215	2.7	218	2.9	218	3.5
Missing	1,002	2.6	204	2.6	225	2.7	221	2.7	202	2.6	150	2.4
Tumour grade												
G1, G2	25,919	67.7	5,550	69.6	5,759	68.9	5,426	67.4	5,098	66.7	4,086	65.7
G3, G4	3,831	10.0	807	10.1	807	9.7	843	10.5	763	10.0	611	9.8
Missing	8,517	22.3	1,620	20.3	1,797	21.5	1,788	22.2	1,788	23.4	1,524	24.5
Emergency presentation												
No	31,507	82.3	6,675	83.7	6,977	83.4	6,689	83.0	6,277	82.1	4,889	78.6
Yes	4210	11.0	685	8.6	795	9.5	869	10.8	924	12.1	937	15.1
Missing	2,550	6.7	617	7.7	591	7.1	499	6.2	448	5.9	395	6.4
Received major resection												
Yes	19,703	51.5	4,333	54.3	4,452	53.2	4,205	52.2	3,871	50.6	2,842	45.7
No	18,564	48.5	3,644	45.7	3,911	46.8	3,852	47.8	3,778	49.4	3,379	54.3

Continued from Supplementary Table 2.

	SES											
	Total		1 (affluent)		2		3		4		5 (deprived)	
	No.	%	No.	%	No.	%	No.	%	No.	%	No.	%
Number of chronic comorbidities												
0	33,858	88.5	7,228	90.6	7,539	90.2	7,128	88.5	6,716	87.8	5,247	84.3
1	3,611	9.4	628	7.9	672	8.0	769	9.5	767	10.0	775	12.5
2+	798	2.1	121	1.5	152	1.8	160	2.0	166	2.2	199	3.2
Number of acute comorbidities												
0	33,942	88.7	7,295	91.5	7,540	90.2	7,148	88.7	6,665	87.1	5,294	85.1
1	3,643	9.5	578	7.3	685	8.2	764	9.5	832	10.9	784	12.6
2+	682	1.8	104	1.3	138	1.7	145	1.8	152	2.0	143	2.3
Obesity at diagnosis	422	1.1	63	0.8	81	1.0	79	1.0	104	1.4	95	1.5

Abbreviations: G, tumour grade; IQR, interquartile range; NA, not applicable; SES, socioeconomic status.

Supplementary Table 3. Hazard Ratios of Death by Bivariable and Multivariable Cox Regression for Colon and Rectal Cancer, Multiple Imputation.

	Colon (n=68,165[b])				Rectum (n=38,267)			
	Bivariable Analysis		Multivariable Analysis		Bivariable analysis		Multivariable analysis	
	HR	95% CI	Adjusted HR[c]	95% CI	HR	95% CI	Adjusted HR[c]	95% CI
SES								
1 (affluent)	Referent		Referent		Referent		Referent	
2	1.08	1.05 to 1.12	1.05	1.00 to 1.09	1.06	1.01 to 1.12	1.01	0.95 to 1.06
3	1.15	1.11 to 1.20	1.10	1.06 to 1.14	1.16	1.10 to 1.22	1.08	1.02 to 1.14
4	1.27	1.23 to 1.32	1.17	1.13 to 1.22	1.27	1.20 to 1.33	1.13	1.07 to 1.19
5 (deprived)	1.33	1.28 to 1.38	1.24	1.19 to 1.30	1.43	1.36 to 1.51	1.22	1.15 to 1.30
Sex								
Male	Referent		Referent		Referent		Referent	
Female	1.08	1.05 to 1.10	0.99	0.97 to 1.02	1.06	1.03 to 1.10	0.97	0.93 to 1.00
Age group								
<65	Referent		Referent		Referent		Referent	
65 to 80	1.31	1.27 to 1.36	1.51	1.45 to 1.56	1.49	1.43 to 1.56	1.64	1.57 to 1.72
80<	2.87	2.78 to 2.96	2.40	2.31 to 2.49	3.73	3.57 to 3.90	3.04	2.89 to 3.19
Year of diagnosis			NA				NA	
2010	Referent				Referent			
2011	1.00	0.97 to 1.03			0.94	0.90 to 0.98		
2012	0.98	0.95 to 1.01			0.87	0.84 to 0.91		
2013	1.04	0.99 to 1.09			0.89	0.83 to 0.96		
Cancer site[a]								
Right-sided colon / Rectosigmoid junction	Referent		Referent		Referent		Referent	
Transverse colon / Rectum	0.99	0.95 to 1.02	1.00	0.96 to 1.04	0.78	0.75 to 0.81	0.81	0.77 to 0.85
Left-sided colon	0.74	0.72 to 0.76	0.79	0.77 to 0.82	NA			
Overlapping or unspecified	2.12	2.05 to 2.20	1.17	1.11 to 1.22	0.93	0.76 to 1.12	0.65	0.52 to 0.82
Stage at diagnosis			NA				NA	
I	Referent				Referent			
II	1.82	1.67 to 1.99			2.13	1.94 to 2.34		
III	3.45	3.17 to 3.75			2.89	2.66 to 3.15		
IV	15.13	13.96 to 16.41			12.34	11.40 to 13.36		
Stage at diagnosis (imputed)								
I	Referent		Referent		Referent		Referent	
II	1.85	1.69 to 2.03	2.02	1.68 to 2.44	2.19	2.00 to 2.39	2.24	1.83 to 2.75
III	3.35	3.09 to 3.64	3.62	3.04 to 4.32	2.85	2.63 to 3.09	2.87	2.39 to 3.45
IV	14.46	13.3 to 15.72	10.55	8.90 to 12.50	11.93	11.01 to 12.93	9.08	7.59 to 10.85

Kajiwara Saito M, *et al. J Epidemiol Community Health* 2021; 75:1155–1164. doi: 10.1136/jech-2021-216754

Continued from Supplementary Table 3.

	Colon (n=68,165[b])				Rectum (n=38,267)			
	Bivariable Analysis		Multivariable Analysis		Bivariable analysis		Multivariable analysis	
	HR	95% CI	Adjusted HR[c]	95% CI	HR	95% CI	Adjusted HR[c]	95% CI
Histology							NA	
Adenocarcinoma	1.00		Referent		Referent			
Non-adenocarcinoma	1.26	1.07 to 1.50	0.75	0.66 to 0.85	0.92	0.83 to 1.02		
Tumour grade			NA				NA	
G1 to G2	Referent				Referent			
G3 to G4	2.19	2.12 to 2.27			2.15	2.05 to 2.26		
Tumour grade (imputed)								
G1 to G2	Referent		Referent		Referent		Referent	
G3 to G4	2.16	2.09 to 2.23	1.57	1.52 to 1.63	2.08	1.98 to 2.18	1.65	1.56 to 1.75
Emergency presentation			NA				NA	
No	Referent				Referent			
Yes	2.21	2.15 to 2.26			3.30	3.16 to 3.44		
Emergency presentation (imputed)								
No	Referent		Referent		Referent		Referent	
Yes	2.18	2.12 to 2.24	1.74	1.69 to 1.79	3.38	3.25 to 3.52	1.93	1.84 to 2.01
Received major resection								
Yes	Referent		Referent		Referent		Referent	
No	5.31	5.18 to 5.43	3.04	2.95 to 3.14	4.82	4.64 to 5.01	2.85	2.72 to 2.97
Number of chronic comorbidities								
0	Referent		Referent		Referent		Referent	
1	1.49	1.45 to 1.54	1.21	1.17 to 1.26	1.62	1.54 to 1.70	1.31	1.24 to 1.38
2+	2.26	2.14 to 2.39	1.41	1.31 to 1.51	2.56	2.35 to 2.79	1.63	1.47 to 1.80
Number of acute comorbidities								
0	Referent		Referent		Referent		Referent	
1	1.56	1.52 to 1.61	1.26	1.22 to 1.30	1.81	1.73 to 1.90	1.34	1.27 to 1.41
2+	2.46	2.33 to 2.59	1.49	1.40 to 1.58	2.84	2.59 to 3.12	1.62	1.46 to 1.81
Obesity at diagnosis			NA				NA	
No	Referent				Referent			
Yes	0.93	0.85 to 1.03			0.92	0.79 to 1.08		

Abbreviations: 95% CI, 95% confidence interval; HR, hazard ratio; NA, not applicable (not included in multivariable model); SES, socioeconomic status. [a]Right-sided colon includes ascending colon, hepatic flexure and caecum. Transverse colon includes transverse colon and splenic flexure. Left-sided colon includes descending colon and sigmoid colon. [b]Information did not match across different datasets in 4 patients for colon cancer therefore excluded. [c]All variables are mutually adjusted. For SES only, adjusted HRs are shown without interaction between SES and stage. For other variables, interaction between SES and stage is adjusted.

Supplementary Table 4. Adjusted Stage-specific Hazard Ratios of Death with Interaction between SES and Stage, Multiple Imputation.

	Colon (n=68,165[a])		Rectum (n=38,267)	
	Adjusted HR[b]	95% CI	Adjusted HR[c]	95% CI
Stage I				
SES 1 (affluent)	Referent		Referent	
2	1.13	0.89 to 1.43	1.05	0.84 to 1.31
3	1.18	0.94 to 1.48	1.08	0.87 to 1.35
4	1.29	1.03 to 1.63	1.21	0.97 to 1.51
5 (deprived)	1.48	1.18 to 1.85	1.38	1.10 to 1.73
Stage II				
SES 1	Referent		Referent	
2	1.08	0.96 to 1.21	1.03	0.88 to 1.21
3	1.16	1.02 to 1.31	1.19	1.02 to 1.39
4	1.38	1.22 to 1.56	1.24	1.05 to 1.46
5	1.45	1.27 to 1.65	1.32	1.12 to 1.56
Stage III				
SES 1	Referent		Referent	
2	1.14	1.05 to 1.24	1.03	0.91 to 1.16
3	1.15	1.04 to 1.26	1.15	1.02 to 1.30
4	1.22	1.12 to 1.33	1.23	1.09 to 1.39
5	1.38	1.25 to 1.51	1.20	1.06 to 1.37
Stage IV				
SES 1	Referent		Referent	
2	1.01	0.96 to 1.07	0.99	0.91 to 1.07
3	1.07	1.02 to 1.13	1.03	0.95 to 1.12
4	1.12	1.06 to 1.17	1.06	0.98 to 1.15
5	1.16	1.09 to 1.23	1.20	1.09 to 1.31

Abbreviations: 95% CI, 95% confidence interval; HR, hazard ratio; SES, socioeconomic status. [a]Information did not match across different datasets in 4 patients for colon cancer therefore excluded. [b]Adjusted for sex, age, site, histology (imputed), tumour grade (imputed), emergency presentation (imputed), receipt of major resection, number of chronic and acute comorbidities. [c]Adjusted for sex, age, site, tumour grade (imputed), emergency presentation (imputed), receipt of major resection, number of chronic and acute comorbidities.

Supplementary Table 5. Point Estimates of Hazard/Excess Hazard Ratios of Death for TVCs for Colon Cancer.

	Overall Survival						Net Survival					
	90 days		6 months		1 year		90 days		6 months		1 year	
	**Adjusted HR**	**95% CI**	**Adjusted HR**	**95% CI**	**Adjusted HR**	**95% CI**	**Adjusted EHR**	**95% CI**	**Adjusted EHR**	**95% CI**	**Adjusted EHR**	**95% CI**
SES	Proportional hazard assumption holds						Proportional hazard assumption holds					
Sex												
Male	Referent		Referent		Referent		Referent		Referent		Referent	
Female	0.96	0.91 to 1.02	0.95	0.89 to 1.01	0.93	0.89 to 0.98	1.00	0.94 to 1.07	0.99	0.91 to 1.08	0.99	0.93 to 1.06
Age group												
<65	Referent		Referent		Referent		Referent		Referent		Referent	
65 to 80	1.64	1.51 to 1.78	1.48	1.35 to 1.61	1.38	1.29 to 1.48	1.54	1.41 to 1.69	1.34	1.18 to 1.51	1.17	1.05 to 1.30
80<	2.57	2.35 to 2.81	2.34	2.12 to 2.57	2.25	2.08 to 2.42	2.10	1.89 to 2.33	1.70	1.44 to 2.02	1.35	1.15 to 1.58
Cancer site[a]												
Right-sided colon	Referent		Referent		Referent		Referent		Referent		Referent	
Transverse colon	1.12	1.03 to 1.22	1.06	0.97 to 1.17	0.99	0.92 to 1.07	1.10	1.00 to 1.21	1.06	0.93 to 1.20	0.97	0.87 to 1.08
Left-sided colon	0.75	0.70 to 0.80	0.71	0.65 to 0.76	0.75	0.71 to 0.80	0.71	0.65 to 0.77	0.63	0.56 to 0.72	0.69	0.64 to 0.74
Overlapping/unspecified	1.20	1.06 to 1.35	1.01	0.86 to 1.19	0.97	0.86 to 1.09	1.16	1.01 to 1.34	0.96	0.76 to 1.20	0.89	0.75 to 1.05
Stage at diagnosis												
I	Referent		Referent		Referent		Referent		Referent		Referent	
II	1.78	1.34 to 2.37	1.80	1.37 to 2.36	1.77	1.39 to 2.26	2.75	1.44 to 5.27	2.70	1.37 to 5.31	3.11	1.60 to 6.04
III	2.50	1.90 to 3.29	3.49	2.69 to 4.54	3.88	3.06 to 4.91	4.90	2.61 to 9.20	7.51	3.90 to 14.48	10.54	5.52 to 20.13
IV	8.40	6.44 to 10.96	12.59	9.76 to 16.25	13.82	11.00 to 17.35	18.56	9.96 to 34.58	31.30	16.33 to 60.00	43.93	23.14 to 83.42
Tumour grade												
G1/G2	Referent		Referent		Referent		Referent		Referent		Referent	
G3/G4	2.01	1.88 to 2.14	2.28	2.13 to 2.45	1.93	1.83 to 2.05	2.17	2.02 to 2.34	2.60	2.33 to 2.90	2.14	1.99 to 2.30
Emergency presentation												
No	Referent		Referent		Referent		Referent		Referent		Referent	
Yes	1.75	1.61 to 1.91	1.83	1.70 to 1.98	1.88	1.78 to 1.99	1.74	1.52 to 1.99	1.91	1.72 to 2.11	2.00	1.86 to 2.16
Received major resection												
Yes	Referent		Referent		Referent		Referent		Referent		Referent	
No	3.91	3.63 to 4.22	3.72	3.46 to 4.00	3.04	2.85 to 3.24	4.30	3.90 to 4.75	4.07	3.69 to 4.47	3.22	2.95 to 3.51
Number of chronic comorbidities	Proportional hazard assumption holds						Proportional hazard assumption holds					
Number of acute comorbidities												
0	Referent		Referent		Referent		Referent		Referent		Referent	
1	1.26	1.16 to 1.36	1.13	1.02 to 1.24	1.15	1.07 to 1.23	1.24	1.13 to 1.37	1.10	0.95 to 1.28	1.13	1.02 to 1.24
2+	1.72	1.51 to 1.95	1.45	1.21 to 1.73	1.45	1.28 to 1.64	1.69	1.45 to 1.99	1.45	1.13 to 1.86	1.44	1.21 to 1.72

Calculated for complete cases only (n=38,070). Interaction between SES and stage is added for the multivariable flexible parametric model. Abbreviations: 95% CI, 95% confidence interval; EHR, excess hazard ratio; HR, hazard ratio; SES, socioeconomic status; TVCs, time-varying covariates. [a]Right-sided colon includes ascending colon, hepatic flexure, and caecum. Transverse colon includes transverse colon and splenic flexure. Left-sided colon includes descending colon and sigmoid colon.

Supplementary Table 6. Point Estimates of Hazard/Excess Hazard Ratios of Death for TVCs for Rectal Cancer.

	Overall survival						Net survival					
	90 days		6 months		1 year		90 days		6 months		1 year	
	Adjusted HR	95% CI	Adjusted HR	95% CI	Adjusted HR	95% CI	Adjusted EHR	95% CI	Adjusted EHR	95% CI	Adjusted EHR	95% CI
SES	Proportional hazard assumption holds						Proportional hazard assumption holds					
Sex	Proportional hazard assumption holds						Proportional hazard assumption holds					
Age group												
<65	Referent		Referent		Referent		Referent		Referent		Referent	
65 to 80	1.85	1.62 to 2.11	1.73	1.56 to 1.92	1.59	1.45 to 1.75	1.73	1.50 to 2.01	1.59	1.41 to 1.78	1.40	1.26 to 1.56
80<	3.08	2.68 to 3.55	3.07	2.74 to 3.44	3.02	2.73 to 3.34	2.43	2.07 to 2.85	2.39	2.09 to 2.73	2.21	1.96 to 2.50
Year of diagnosis	Proportional hazard assumption holds						Proportional hazard assumption holds					
Cancer site												
Rectosigmoid junction	Referent		Referent		Referent		Referent		Referent		Referent	
Rectum	0.82	0.73 to 0.93	0.88	0.80 to 0.98	0.91	0.83 to 1.00	0.80	0.70 to 0.92	0.85	0.75 to 0.96	0.86	0.78 to 0.96
Overlapping/unspecified	0.48	0.12 to 1.96	0.78	0.30 to 2.00	0.61	0.27 to 1.35	0.39	0.07 to 2.23	0.71	0.24 to 2.14	0.61	0.25 to 1.51
Stage at diagnosis	Proportional hazard assumption holds						Proportional hazard assumption holds					
Tumour grade												
G1/G2	Referent		Referent		Referent		Referent		Referent		Referent	
G3/G4	2.01	1.79 to 2.24	2.21	2.01 to 2.42	1.97	1.80 to 2.16	2.15	1.90 to 2.43	2.38	2.14 to 2.64	2.14	1.94 to 2.37
Emergency presentation												
No	Referent		Referent		Referent		Referent		Referent		Referent	
Yes	1.64	1.40 to 1.92	1.57	1.38 to 1.79	1.62	1.47 to 1.79	1.58	1.29 to 1.95	1.64	1.41 to 1.90	1.76	1.57 to 1.97
Received major resection	Proportional hazard assumption holds						Proportional hazard assumption holds					
Number of chronic comorbidities	Proportional hazard assumption holds						Proportional hazard assumption holds					
Number of acute comorbidities	Proportional hazard assumption holds						Proportional hazard assumption holds					

Calculated for complete cases only (n=22,631). Interaction between SES and stage is added for the multivariable flexible parametric model. Abbreviations: 95% CI, 95% confidence interval; EHR, excess hazard ratio; HR, hazard ratio; SES, socioeconomic status; TVCs, time-varying covariates.

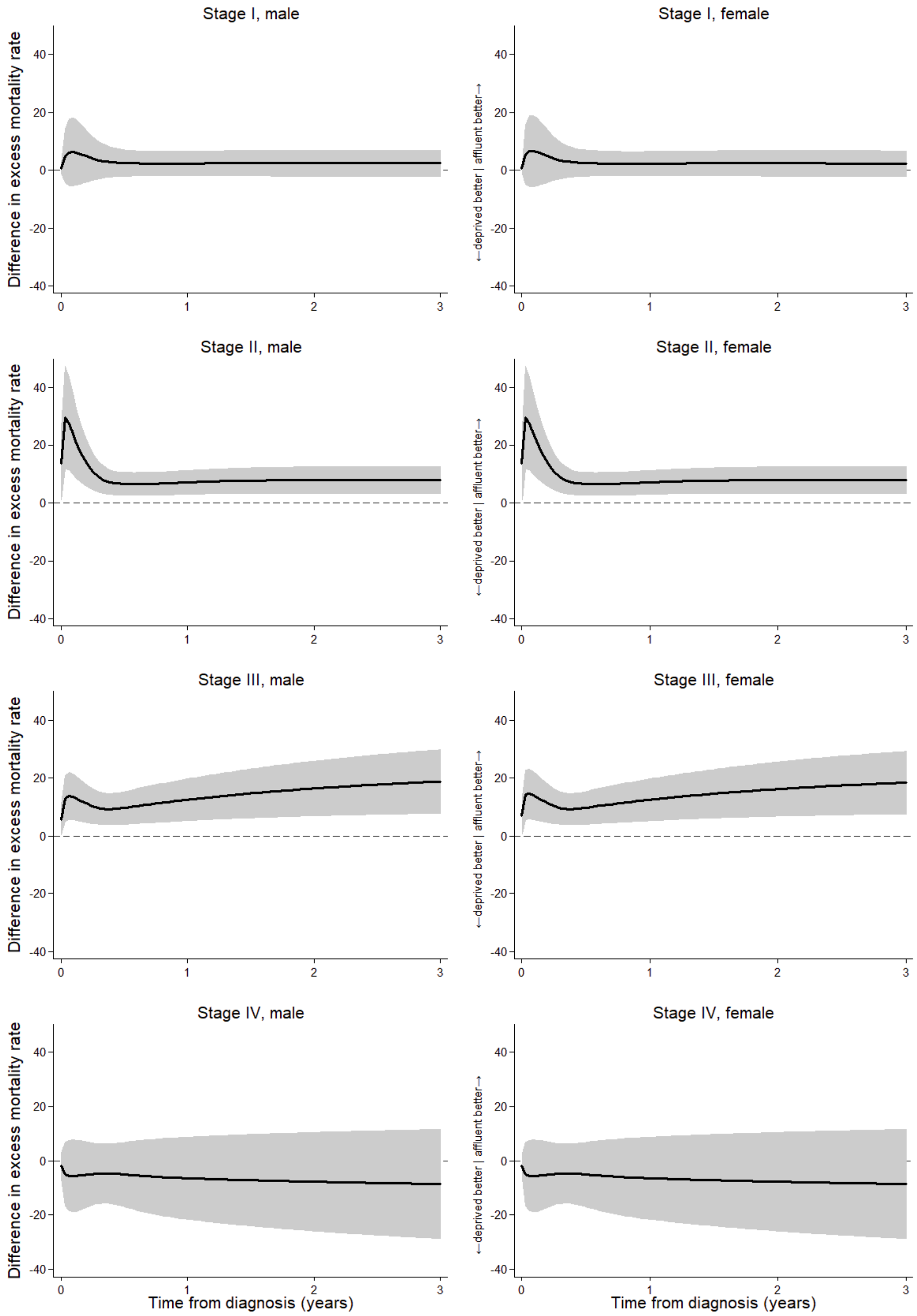

Supplementary Figure 1. Difference in Excess Hazard of Death between SES 1 (the Most Affluent) and SES 5 (the Most Deprived), Colon Cancer.

Per 1000 PYs (person-years). Abbreviations: SES, socioeconomic status. Age group was set at under 65 years old, cancer site at right-sided colon, tumour grade at G1/G2, no emergency presentation, received major resection and no chronic or acute comorbidities.

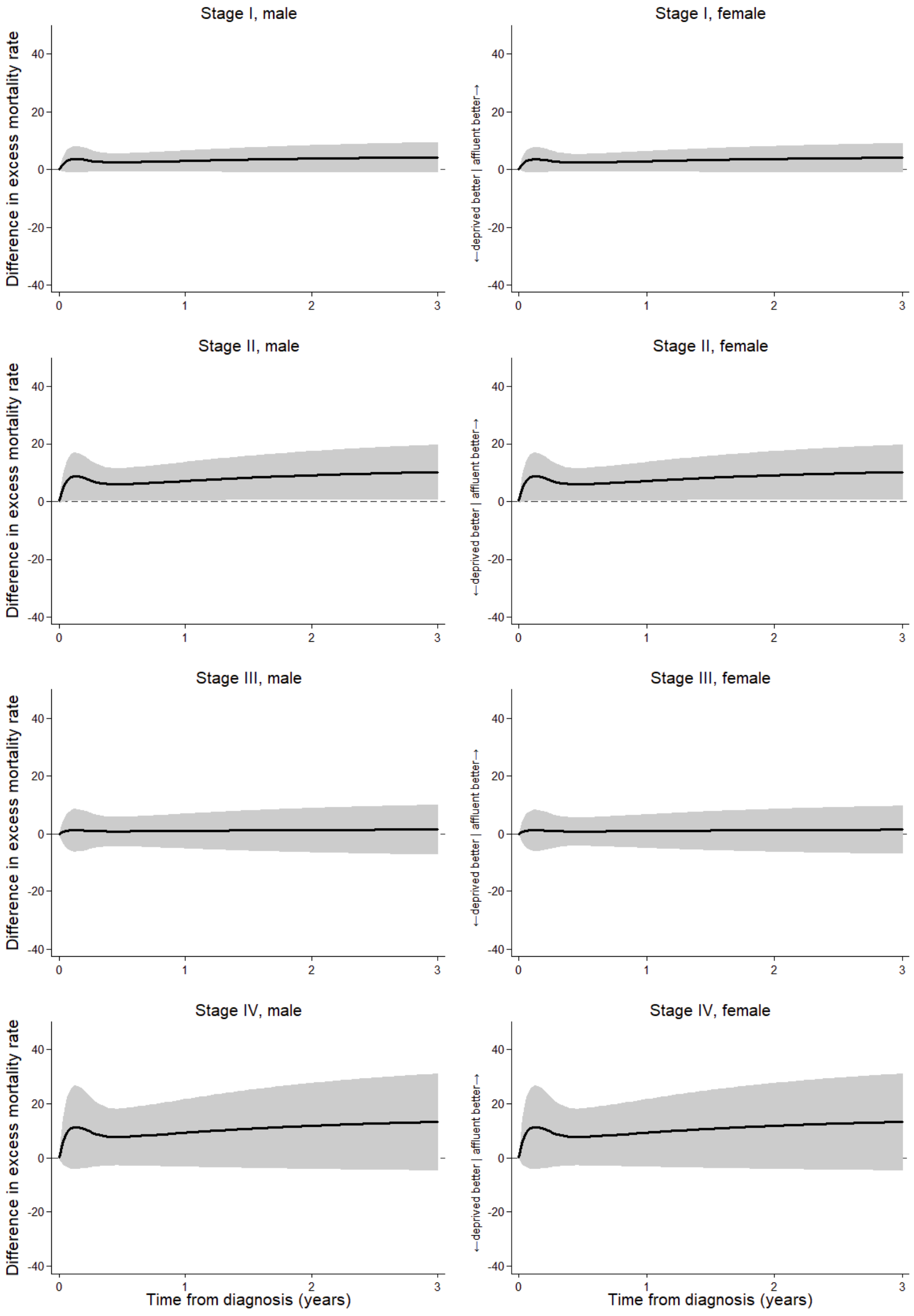

Supplementary Figure 2. Difference in Excess Hazard of Death between SES 1 (the Most Affluent) and SES 5 (the Most Deprived), Rectal Cancer.

Per 1000 PYs (person-years). Abbreviations: SES, socioeconomic status. Age group was set at under 65 years old, cancer site at rectosigmoid junction, tumour grade at G1/G2, no emergency presentation, received major resection and no chronic or acute comorbidities. Year of diagnosis was set at 2010.

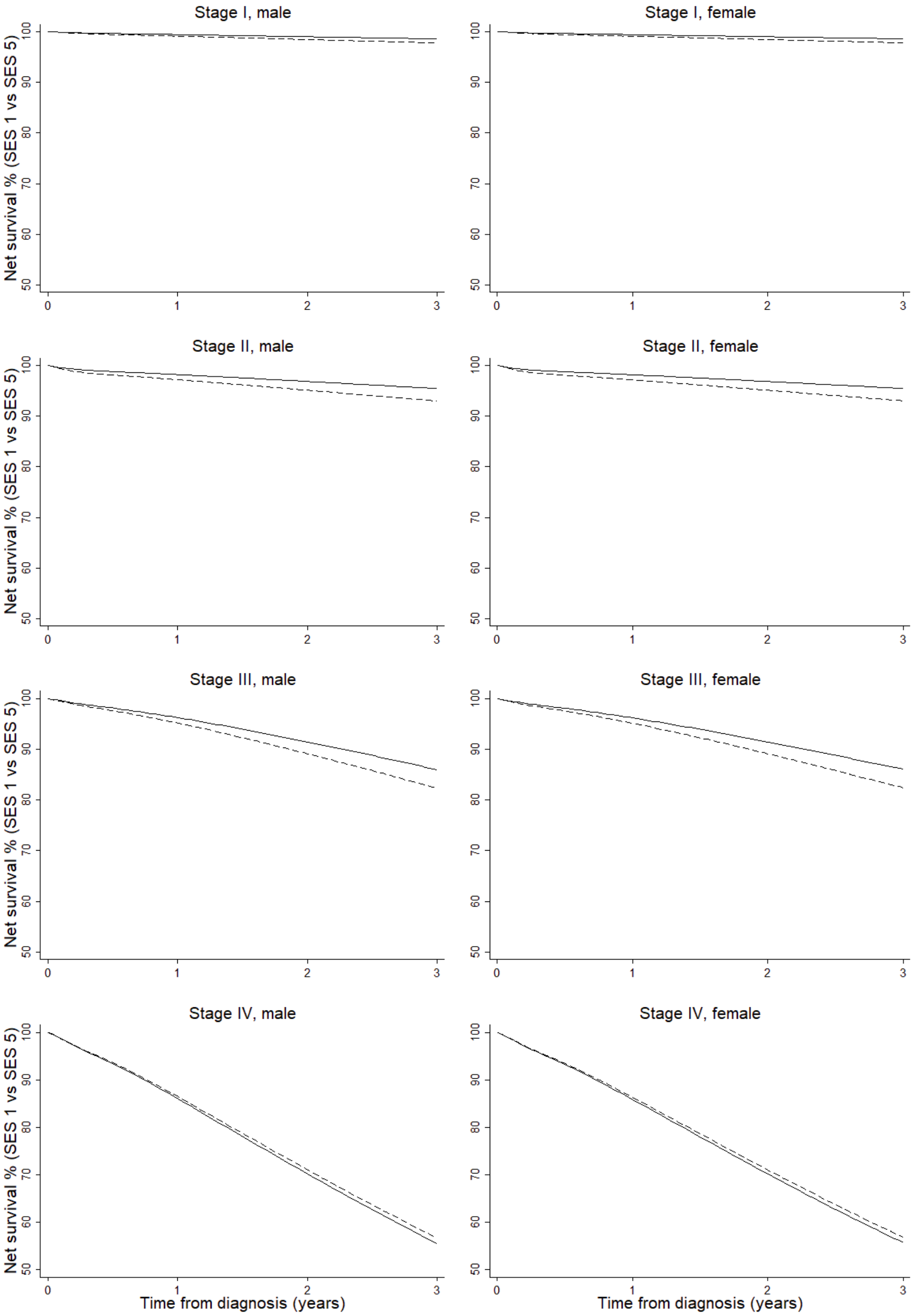

Supplementary Figure 3. Net Survival Curves of SES 1 (Solid Line) and SES 5 (Dotted Line), Colon Cancer.

Abbreviations: SES, socioeconomic status.

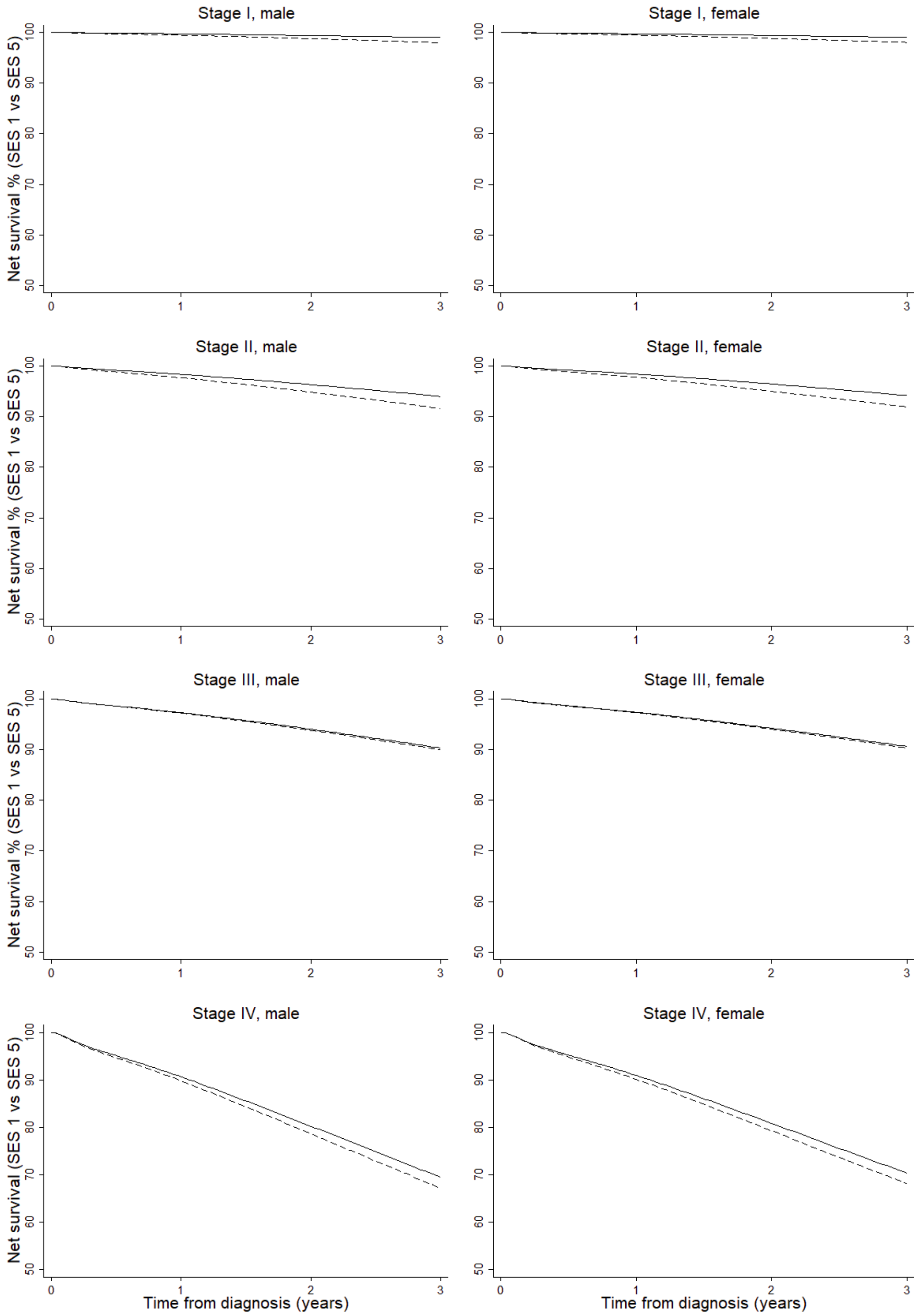

Supplementary Figure 4. Net Survival Curves of SES 1 (Solid line) and SES 5 (Dotted line), Rectal Cancer.

Abbreviations: SES, socioeconomic status.

Kajiwara Saito M, *et al. J Epidemiol Community Health* 2021; 75:1155–1164. doi: 10.1136/jech-2021-216754
